# Statin use associated with a reduced risk of pneumonia requiring hospitalization in patients with myocardial infarction: a nested case-control study

**DOI:** 10.1186/s12872-016-0202-x

**Published:** 2016-01-28

**Authors:** Chao-Feng Lin, Ya-Hui Chang, Ju-Chi Liu, Ming-Tsang Chuang, Li-Nien Chien

**Affiliations:** Division of Cardiology, Department of Internal Medicine, Shuang Ho Hospital, Taipei Medical University, New Taipei City, Taiwan; Pharmacy Department of Mackay Memorial Hospital, Taipei, Taiwan; School of Public Health, College of Public Health and Nutrition, Taipei Medical University, Taipei, Taiwan; School of Health Care Administration, College of Management, Taipei Medical University, Taipei, Taiwan; Division of Cardiology, Department of Internal Medicine, MacKay Memorial Hospital, Taipei, Taiwan; Department of Medicine, Mackay Medical College, New Taipei, Taiwan

**Keywords:** Myocardial infarction, Pneumonia, Statins, Incidence-density sampling

## Abstract

**Background:**

Statins have been reported to prevent adverse cardiovascular events in patients with myocardial infarction (MI). However, the association of statin use and the risk of pneumonia requiring hospitalization in MI patients remains unclear.

**Methods:**

A nested case-control study was conducted by using data from the National Health Insurance Research Database of Taiwan. Among 24,975 patients with MI, 2686 case patients with pneumonia requiring hospitalization were age- and sex-matched with 10,726 control patients using the incidence density sampling approach. Duration and dosage of statin use were obtained from pharmaceutical claims. Conditional logistic regression analyses were used to estimate the risk of hospitalization for pneumonia associated with statin use adjusted for patient’s demographics, medical conditions and prescribed medications.

**Results:**

Statin use was associated with a 15 % reduced risk of pneumonia requiring hospitalization among MI patients (adjusted odds ratio [aOR] = 0.85, 95 % confidence interval [CI] = 0.77–0.95, *P* = 0.004). The association was more significant for MI patients unexposed to statin pretreatment (aOR = 0.76, 95 % CI = 0.64–0.90, *P* = 0.001). Statins also exhibited favorable benefits in a time- and dose-dependent manner. The results were consistent in various subgroup analysis of the patients who were female, age ≥ 65 years, a low CHADS_2_ (i.e. congestive heart failure, hypertension, diabetes mellitus, previous stroke and age > 75 years old) score, and fewer comorbidities. Atorvastatin, fluvastatin and simvastatin were the most common prescribed statins and had similar effects.

**Conclusions:**

Statins might be considered as an adjunctive therapy to reduce the risk of hospitalization for pneumonia for MI patients under thorough evaluation of individual comorbidities, previous statin use and optimal dosage.

**Electronic supplementary material:**

The online version of this article (doi:10.1186/s12872-016-0202-x) contains supplementary material, which is available to authorized users.

## Background

Myocardial infarction (MI) is the most acute and severe presentation of ischemic heart disease. Approximately 620,000 people have a new coronary attack each year in the United States, and 15 % of those do not survive the event [1, 2]. Besides, survivors of MI have a higher chance of illness and death from cardiac or noncardiac causes compared with the general population [1–3]. Patients with MI had a 79 % increased risk of incident infection compared with those without an MI history, and the most frequent infection was pneumonia [3]. Additionally, MI patients had a significantly higher rate of all-cause mortality during the course of pneumonia than that of patients without a history of MI [4]. Therefore, strategies for preventing the development of adverse cardiovascular events and incident pneumonia among patients with MI are crucial.

Statins are competitive inhibitors of 3-hydroxy-3-methylglutaryl-coenzyme A (HMG-CoA) reductase, a rate-limiting enzyme of the cholesterol biosynthesis pathway [5], and are commonly used in the primary and secondary prevention of atherosclerotic cardiovascular diseases [6]. Previous researches have also demonstrated that statin pretreatment improves the clinical outcomes of MI [7, 8]. Recently, epidemiological studies have shown that statins have pleiotropic effects, resulting in a reduced risk of incident pneumonia in the general population as well as patients with diabetes [9–12]. However, whether statin use is associated with a reduced risk of pneumonia in MI patients who are at high cardiovascular risk and susceptible to pneumonia remains unclear.

The aim of this study was to investigate the association between statin use and pneumonia hospitalization among patients with a history of MI. We conducted a nested case–control study by using data from the National Health Insurance Research Database (NHIRD) of Taiwan. Subgroup analysis was performed to identify the aforementioned association among patients with different characteristics and those who were unexposed to statin pretreatment.

## Methods

### Ethics statement

This retrospective observational research was approved by the Joint Institutional Review Board of Taipei Medical University (TMU-JIRB No. 201404055). Data from the NHIRD was provided by the National Health Insurance Administration (NHIA) of Taiwan that covers 99 % of residents in Taiwan under the legislation of National Health Insurance (NHI). To protect the privacy of beneficiaries, individual identifiers have been encrypted before data are released to researchers. Consequently, informed consent of the participants was exempted under the full review process of the Joint Institutional Review Board of Taipei Medical University.

### Study design and data source

This nested case-control study was conducted by using data from NHIRD which were collected between 2000 and 2011. The NHIRD files contained International Classification of Diseases, Ninth Revision, Clinical Modification (ICD-9-CM) disease diagnosis codes, treatment procedures, date of service, reimbursement amounts, demographic information on beneficiaries and beneficiary- and provider-encrypted identifiers. Claims for prescribed drugs were also provided and could be classified according to the Anatomical Therapeutic Chemical (ATC) classification system. To verify the accuracy of diagnoses and the rationale for treatments, the NHIA routinely samples a proportion of the NHI claims. Additionally, hospitals and clinics are penalized if they provide any unnecessary medical treatment to patients [13].

### Study cohort

**Fig. 1:**
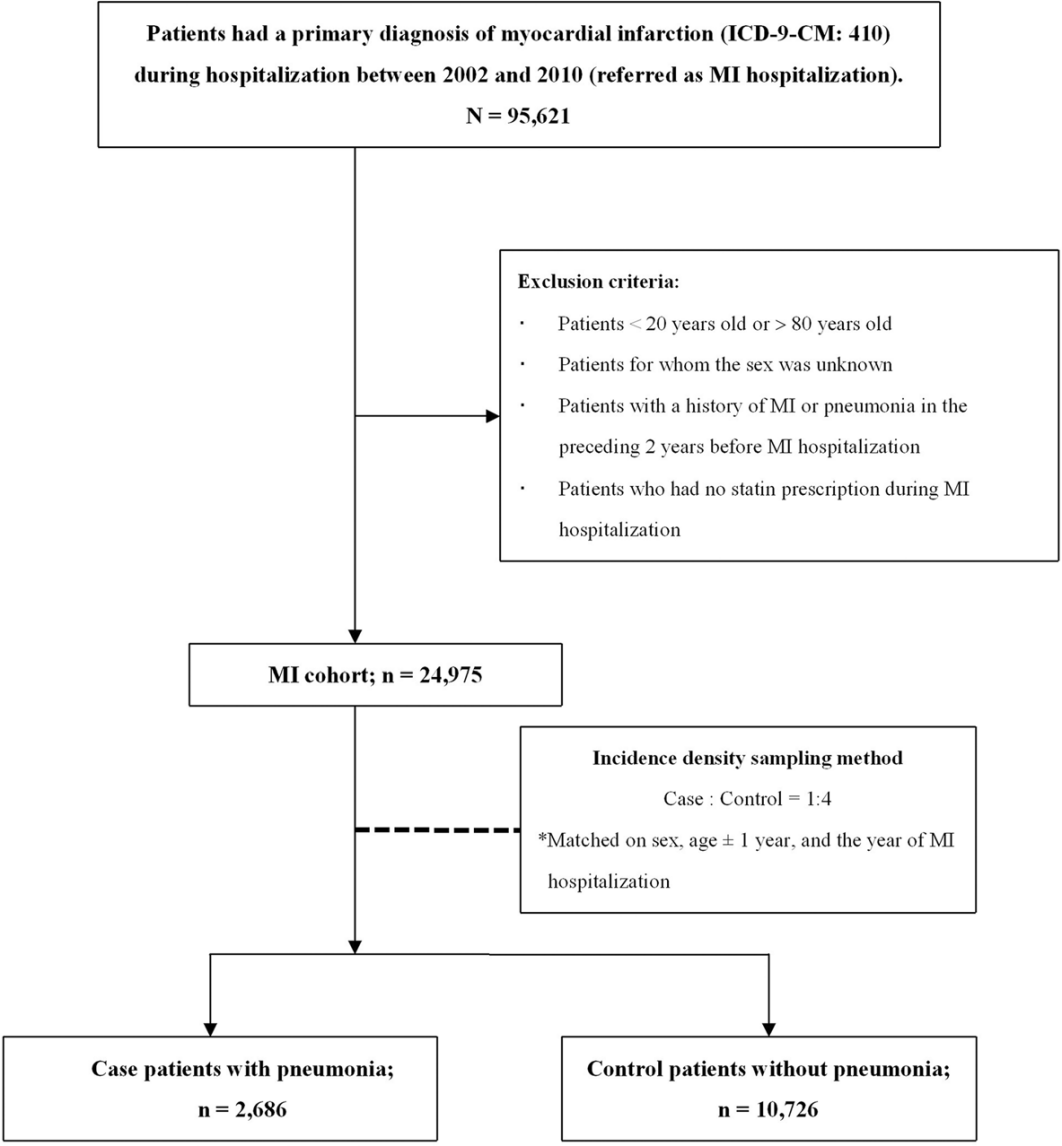
Flow diagram of patient selection. MI = myocardial infarction

The study cohort consisted of patients who were first admitted for a primary diagnosis of MI (ICD-9-CM: 410) between the year of 2002 and 2010, and received prescriptions of statins during MI hospitalization. We excluded participants who were < 20 years old or > 80 years old, for whom the sex was unidentified, and had a history of MI or pneumonia (ICD-9-CM: 480–486) in the preceding 2 years. Figure 1 presents the patient selection process.

### Case ascertainment and control selection

The case patients were those who were readmitted for the primary diagnosis of incident pneumonia (ICD-9-CM: 480–486) requiring hospitalization after MI admission. In Taiwan, the diagnosis of pneumonia at least must comply with the positive findings of the patient’s chest X-ray. The date of hospitalization for pneumonia was defined as the index date. The control patients were randomly selected by using incidence density sampling of all patients with MI who were alive and free of pneumonia at the time of being observed. Each case patient was matched to 4 control patients by sex, age ± 1 year, year of hospitalization for MI, and follow-up period. The control patients were assigned a pseudo index date of pneumonia admission, which corresponded to the index date of their matched cases.

### Statin exposure

Statin use was determined based on prescription claims in the NHIRD. The duration of statin use was defined as the period between the latest date of prescription and the index date of pneumonia of case patients or the pseudo index date of control patients (hereafter, “index date”). For each patient, the drug prescriptions were traced back from the index date up to 1 year. Both case and control patients were categorized into “users” if they had any statin claim and “nonusers” if they had no statin claims 1 year before the index date. Subsequently, the users were further classified into “current users” for 0–90 days, “recent users” for 91–180 days, or “former users” for >180 days. The dose-response effect was assessed based on the defined daily dose (DDD), which is the assumed maintenance dose per day for adults recommended by the World Health Organization [14], and was classified as < 0.5, 0.5–1.0, and > 1.0 of the DDD within a 90-day period (DDD_90_). Additionally, we performed analyses to compare the effects of different statins among the patients with statin prescription within a 90-day period before the index date. A specific statin was defined if it was the nearest and longest statin to the index date. The DDDs of the statins considered in our study are provided in Additional file 1: Table S1.

### Potential confounding variables

Previous or coexisting medical conditions were recorded if the patients had ≥ 2 diagnostic claims for diabetes mellitus (DM), hypertension (HTN), congestive heart failure (CHF), stroke, asthma, chronic obstructive pulmonary disease (COPD), chronic kidney disease (CKD), chronic liver disease (CLD), Parkinson disease or dementia. We also considered and controlled for the CHADS_2_ score, which is the sum of the risk factors for CHF, HTN, DM and stroke as well as the age > 75 years at the diagnosis of MI. The CHADS_2_ score was initially used to predict the risk of stroke in patients with nonvalvular atrial fibrillation [15], and has been proposed recently for predicting the prognosis of patients with acute coronary syndrome [16, 17]. The history of influenza and pneumonia vaccination and prescribed medications before the index date were further adjusted. For each patient, the records of medical conditions and medications were traced from the index date up to 1 year. Detailed information on ICD-9-CM diagnosis codes and the ATC classification system codes are provided in Additional file 2: Table S2 and Additional file 3: Table S3, respectively.

### Statistical analyses

The primary analysis was to examine the association between statin use and the risk of pneumonia requiring hospitalization. To investigate the effect of statin pretreatment before an MI event, the secondary analysis was limited to MI patients unexposed to statin pretreatment. The stratified analysis of the patients with a specific characteristic was also performed. For all variables of interest, risk estimates were computed as both univariate and multivariate analyses, with additional adjustments for potential confounders. Conditional logistic regression was used to estimate unadjusted and adjusted odds ratios (ORs) and 95 % confidence intervals (CIs) for the association of statin exposure and the risk of hospitalization for pneumonia; and the incidence density sampling yields ORs that are interpretable as unbiased estimates of the incidence ORs [18, 19]. All analyses were performed using SAS/STAT 9.3 (SAS Institute Inc., Cary, NC, USA) and STATA 12 (Stata Corp LP, College Station, TX, USA); *P* < 0.05 was considered significant.

## Results

Of the 24,975 patients with a history of MI, we identified 2686 case patients who were hospitalized for pneumonia and 10,726 control patients who were not hospitalized for pneumonia (Fig. 1). Compared with the control patients, the case patients had higher CHADS_2_ scores (CHADS_2_ scores ≥ 1: case 36.6 %; control 17.7 %), and higher rates of previous or coexisting DM, HTN, CHF, stroke, asthma, COPD, CKD, CLD and dementia. In addition, the case patients were more likely to use antineoplastic drugs, proton pump inhibitors (PPIs), steroids, angiotensin converting enzyme inhibitor and angiotensin receptor blockade (ACEI/ARBs), immunosuppressants, immunostimulants and nitrates compared with the control patients (Table 1).

**Table 1:** Baseline characteristics of case patients with pneumonia and control patients

		Controls		Cases				
Variables		N	(%)	N	(%)	Crude OR	(95 % CI)	*P*
Sample size		10,726	(100.0)	2,686	(100.0)			
Female		3,280	(30.6)	824	(30.7)			
Age (y)	Mean (± SD)	67.1	(±10.0)	67.1	(±10.0)			
CHADS_2_ score								
0		8,830	(82.3)	1,703	(63.4)	1.00	(Ref.)	
1–2		1,695	(15.8)	838	(31.2)	3.33	(2.99–3.71)	<0.001
> 2		201	(1.9)	145	(5.4)	5.85	(5.03–6.79)	<0.001
Comorbidities, yes (Ref. = No)								
Diabetes mellitus		800	(7.5)	552	(20.6)	3.33	(2.94–3.76)	<0.001
Hypertension		418	(3.9)	268	(10)	2.77	(2.36–3.26)	<0.001
Congestive heart failure		800	(7.5)	570	(21.2)	3.43	(3.04–3.87)	<0.001
Stroke		1,372	(12.8)	654	(24.3)	2.21	(1.98–2.45)	<0.001
Asthma		133	(1.2)	74	(2.8)	2.28	(1.70–3.04)	<0.001
COPD		246	(2.3)	196	(7.3)	3.43	(2.82–4.18)	<0.001
Chronic kidney disease		118	(1.1)	108	(4)	3.76	(2.88–4.91)	<0.001
Chronic liver disease		28	(0.3)	16	(0.6)	2.29	(1.23–4.22)	0.008
Parkinson disease		20	(0.2)	8	(0.3)	1.60	(0.70–3.63)	0.260
Dementia		49	(0.5)	24	(0.9)	1.96	(1.20–3.19)	0.007
Medication use, yes (Ref. = No)								
Antineoplastic drug		67	(0.6)	71	(2.6)	4.35	(3.10–6.10)	<0.001
PPI		1,127	(10.5)	665	(24.8)	2.86	(2.56–3.19)	<0.001
Steroid		1,756	(16.4)	736	(27.4)	1.96	(1.77–2.16)	<0.001
ACEI/ARB		5,983	(55.8)	1,674	(62.3)	1.33	(1.21–1.45)	<0.001
Antiviral drug		143	(1.3)	47	(1.7)	1.32	(0.95–1.84)	0.100
Immunosuppressants		19	(0.2)	12	(0.4)	2.53	(1.22–5.20)	0.010
Immuostimulants		7	(0.1)	8	(0.3)	5.01	(1.72–14.5)	0.003
Nitrate		6,277	(58.5)	1,847	(68.8)	1.62	(1.47–1.78)	<0.001
Antiplatelet		9,151	(85.3)	2,330	(86.7)	1.13	(0.99–1.28)	0.050
Vaccine (influenza or pneumococcal)		2,551	(23.8)	631	(22.8)	0.94	(0.85–1.05)	0.280

*ACEI/ARB* angiotensin-converting enzyme inhibitor/angiotensin receptor blockade, *CHADS*_*2*_ congestive heart failure, hypertension, age > 75 years, diabetes, previous stroke, *CI* confidence interval, *COPD* chronic obstructive pulmonary disease, *OR* odds ratio, *PPI* proton pump inhibitor, *Ref* reference group, *SD* standard deviation

The percentage of any use of statins among the case and control patients was 68.9 and 71.6 %, respectively. Statin use was associated with a 15 % reduced risk of incident pneumonia requiring hospitalization after we controlled for all risk factors (aOR 0.85, 95 % CI 0.77–0.95, *P* = 0.004) (Table 2). Additionally, the timing of statin use for the case patients significantly differed from that for the control patients (Table 2). The odds of current users for the case patients were lower than that for the control patients (aOR 0.75, 95 % CI 0.67–0.85, *P* < 0.001). We also observed that the DDD_90_ was associated with a dose-dependent manner that the benefits of statin to prevent the occurrence of pneumonia were determined when the DDD_90_ was ≥ 0.5. However, the protective effect of a DDD_90_ of 0.5–1.0 (aOR 0.75, 95 % CI 0.66–0.84, *P* < 0.001) was closed to that of a DDD_90_ ≥ 1.0 (aOR 0.74, 95 % CI 0.63–0.87, *P* = 0.002) (Table 2).

**Table 2:** Association between statin use and the risk of pneumonia requiring hospitalization

	Controls		Cases		Crude OR		Adjusted^a^ OR	
	N	(%)	N	(%)	(95 % CI)	*P*	(95 % CI)	*P*
Sample size	10,726	(100.0)	2,686	(100.0)	-		-	
Statin measurement								
Any statin ≤ 365 d								
Non-user	3,041	(28.4)	835	(31.1)	1.00 (Ref.)		1.00 (Ref.)	
User	7,685	(71.6)	1,851	(68.9)	0.87 (0.78–0.95)	0.003	0.85 (0.77–0.95)	0.004
By recency								
Non-user	3,041	(28.4)	835	(31.1)	1.00 (Ref.)		1.00 (Ref.)	
≤ 90 d (current)	4,778	(44.5)	963	(35.9)	0.72 (0.64–0.80)	<0.001	0.75 (0.67–0.85)	<0.001
91–180 d (recent)	697	(6.5)	229	(8.5)	1.17 (0.98–1.39)	0.080	1.06 (0.87–1.28)	0.570
>180 d (former)	2,210	(20.6)	659	(24.5)	1.08 (0.96–1.23)	0.190	0.98 (0.86–1.12)	0.810
By DDD_90_								
Non-user	3,041	(28.4)	835	(31.1)	1.00 (Ref.)		1.00 (Ref.)	
≤ 0.5 DDD_90_	1,278	(11.9)	388	(14.4)	1.05 (0.92–1.19)	0.510	0.98 (0.85–1.13)	0.760
0.5-1 DDD_90_	2,808	(26.2)	539	(20.1)	0.66 (0.59–0.74)	<0.001	0.75 (0.66–0.84)	<0.001
> 1 DDD_90_	1,389	(12.9)	265	(9.9)	0.66 (0.57–0.76)	<0.001	0.74 (0.63–0.87)	0.002

*ACEI/ARB* angiotensin-converting enzyme inhibitor/angiotensin receptor blockade, *CHADS*_*2*_ congestive heart failure, hypertension, age > 75 years, diabetes, previous stroke, *CI* confidence interval, *COPD* chronic obstructive pulmonary disease, *DDD* defined daily dose, *DDD*_*90*_ average defined daily dose within 90 days, *OR* odds ratio, *PPI* proton pump inhibitor, *Ref* reference group, *SD* standard deviation

^a^ Adjusted for CHASD_2_ score, medical conditions (diabetes mellitus, hypertension, chronic heart failure, stroke, COPD, chronic kidney disease, chronic liver disease, Parkinson disease, and dementia) and medication use (antineoplastic drug, PPI, ACEI/ARB, antiviral drug, immunosuppressants, immunostimulants, nitrate, antiplatelet and influenza or pneumococcal vaccine)

Of the patients who were unexposed to statin pretreatment at the time of being diagnosed with MI, we identified 1313 case patients who were hospitalized for pneumonia and 6321 control patients who were not hospitalized for pneumonia. The percentage of any use of statins among the case and control patients was 60.4 and 66.3 %, respectively. Among patients unexposed to pretreatment, statin users had a 24 % decreased risk of pneumonia hospitalization (aOR 0.76, 95 % CI 0.64–0.90, *P* = 0.001). The results of current users and dosage manner were consistent with the findings of the primary analysis, but the association was stronger (Table 3).

**Table 3:** Subgroup analysis of patients unexposed to statin pretreatment

	Controls		Cases		Crude OR		Adjusted^a^ OR	
	N	(%)	N	(%)	(95 % CI)	*P*	(95 % CI)	*P*
Sample size	6,321	(100.0)	1,313	(100.0)	-		-	-
Statin Measurement								
Any statin ≤ 365 d								
Non-user	2,130	(33.7)	520	(39.6)	1.00 (Ref.)		1.00 (Ref.)	
User	4,191	(66.3)	793	(60.4)	0.75 (0.65–0.87)	<0.001	0.76 (0.64–0.90)	0.001
By recency								
Non-user	2,130	(33.7)	520	(39.6)	1.00 (Ref.)		1.00 (Ref.)	
≤ 90 d (current)	2,683	(42.4)	403	(30.7)	0.60 (0.51–0.71)	<0.001	0.65 (0.54–0.79)	<0.001
91–180 d (recent)	376	(5.9)	110	(8.4)	1.16 (0.88–1.52)	0.290	1.20 (0.88–1.64)	0.240
> 180 d (former)	1,132	(17.9)	280	(21.3)	0.97 (0.80–1.18)	0.770	0.84 (0.68–1.05)	0.130
By DDD_90_								
Non-user	2,130	(33.7)	520	(39.6)	1.00 (Ref.)		1.00 (Ref.)	
≤ 0.5 DDD_90_	757	(12.0)	178	(13.6)	0.99 (0.80–1.22)	0.900	0.99 (0.78–1.27)	0.960
0.5–1 DDD_90_	1,574	(24.9)	233	(17.7)	0.61 (0.51–0.73)	<0.001	0.73 (0.59–0.89)	0.002
> 1 DDD_90_	728	(11.5)	102	(7.8)	0.55 (0.43–0.69)	<0.001	0.65 (0.50–0.85)	0.001

*ACEI/ARB* angiotensin-converting enzyme inhibitor/angiotensin receptor blockade, *CHADS*_*2*_ congestive heart failure, hypertension, age > 75 years, diabetes, previous stroke, *CI* confidence interval, *COPD* chronic obstructive pulmonary disease, *DDD* defined daily dose, *DDD*_*90*_ average defined daily dose within 90 days, *OR* odds ratio, *PPI* proton pump inhibitor, *Ref* reference group, *SD* standard deviation

^a^ Adjusted for CHASD_2_ score, medical conditions (diabetes mellitus, hypertension, chronic heart failure, stroke, COPD, chronic kidney disease, chronic liver disease, Parkinson disease, and dementia) and medication use (antineoplastic drug, PPI, ACEI/ARB, antiviral drug, immunosuppressants, immunostimulants, nitrate, antiplatelet and influenza or pneumococcal vaccine)

**Fig. 2:**
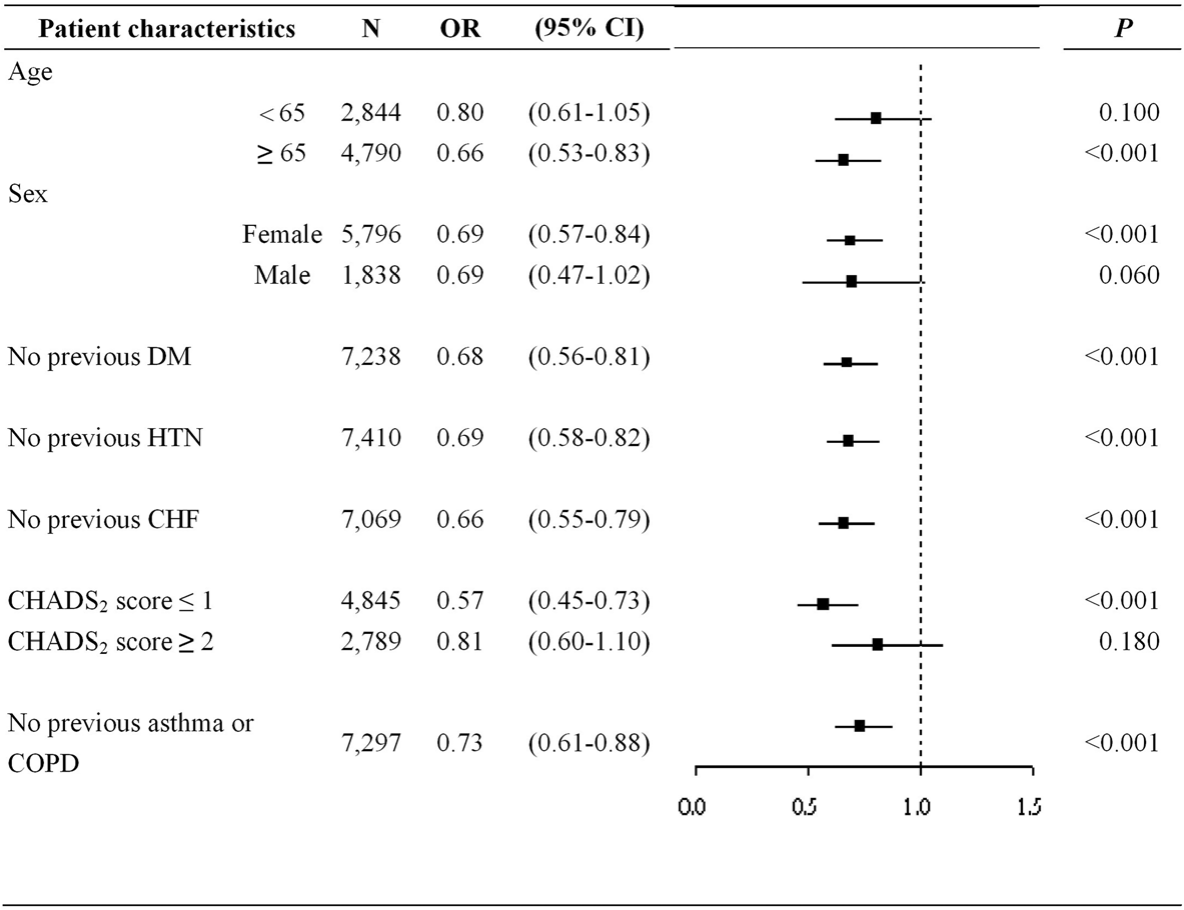
Subgroup analysis of patients in different comorbidity subgroups. CHADS_2_ = congestive heart failure, hypertension, age > 75 years, diabetes mellitus and previous stroke; CHF = congestive heart failure; CI = confidence interval; COPD = chronic obstructive pulmonary disease; DM = diabetes mellitus; HTN = hypertension; OR = odds ratio

We performed a stratified analysis and redefined “statin users” if patients had a statin of DDD_90_ ≥ 0.5. Our results showed a negative association between statin exposure and the risk of pneumonia requiring hospitalization in the patients who were female, were aged ≥ 65 years, had lower CHADS_2_ scores (0 or 1), or had no history of DM, HTN, CHF, asthma or COPD (Fig. 2).

The major statins used within a 90-day period prior to the index date were atorvastatin, fluvastatin and simvastatin (Table 4). For all patients with MI, fluvastatin and simvastatin had similar associations with the risk of pneumonia requiring hospitalization compared to atorvastatin (fluvastatin: aOR 1.14, 95 % CI 0.85–1.52, *P* = 0.390; simvastatin: aOR 1.19, 95 % CI 0.87–1.64, *P* = 0.283). For patients unexposed to statin pretreatment, the results were consistent with the findings of overall MI patients.

**Table 4:** Statin type and the risk of pneumonia requiring hospitalization within a 90-day exposure

	Controls	Cases	Crude OR		Adjusted^a^ OR	
	N	N	(95 % CI)	*P*	(95 % CI)	*P*
Overall MI patients	4,778	963	-		-	
Statin type						
Artovastatin	3,242	639	1.00 (Ref.)		1.00 (Ref.)	
Fluvastatin	556	132	1.21 (0.94–1.56)	0.136	1.14 (0.85–1.52)	0.390
Simvastatin	510	109	1.04 (0.79–1.38)	0.781	1.19 (0.87–1.64)	0.283
Other	470	83	0.87 (0.65–1.17)	0.363	0.73 (0.51–1.03)	0.075
MI patients unexposed to statin pretreatment	2,683	403				
Statin type						
Atorvastatin	1,863	270	1.00 (Ref.)		1.00 (Ref.)	
Fluvastatin	297	55	1.16 (0.71–1.90)	0.549	1.11 (0.6–2.04)	0.743
Simvastatin	278	54	0.80 (0.49–1.32)	0.386	1.12 (0.59–2.10)	0.729
Other	245	24	0.65 (0.36–1.17)	0.153	0.66 (0.31–1.41)	0.286

*ACEI/ARB* angiotensin-converting enzyme inhibitor/angiotensin receptor blockade, *CHADS*_*2*_ congestive heart failure, hypertension, age > 75 years, diabetes, previous stroke, *CI* confidence interval; COPD, chronic obstructive pulmonary disease, *DDD* defined daily dose, *DDD*_*90*_ average defined daily dose within 90 days, *OR* odds ratio, *PPI* proton pump inhibitor, *Ref* reference group, *SD* standard deviation

^a^ Adjusted for CHASD_2_ score, medical conditions (diabetes mellitus, hypertension, chronic heart failure, stroke, COPD, chronic kidney disease, chronic liver disease, Parkinson disease, and dementia) and medication use (antineoplastic drug, PPI, ACEI/ARB, antiviral drug, immunosuppressants, immunostimulants, nitrate, antiplatelet and influenza or pneumococcal vaccine)

## Discussion

In this nested case-control study, we observed that statin use in MI patients was associated with a 15-25 % reduced risk of pneumonia requiring hospitalization. We also observed that the benefits of statins were particularly marked for current users who used statins within 90 days and for those who used statins of ≥ 0.5 DDD_90_. In addition, the favorable effect was shown in the patients who were female, were aged ≥ 65 years, and had no history of cardiovascular and respiratory diseases. Atorvastatin, fluvastatin and simvastatin were the most common prescribed statins and had similar effects on reducing the risk of pneumonia requiring hospitalization.

The effect of statin use on reducing the occurrence of pneumonia has been shown in the general population [10, 11]. In this present study, we additionally found that statin use might also have a favorable effect on decreasing pneumonia hospitalization in MI patients who were more susceptible to pneumonia and had more comorbidities compared to general population. However, this favorable effect was not significant when the analysis was limited to MI patients with a higher CHADS_2_ score (CHADS_2_ score ≥ 2). Some unmeasured differences in baseline characteristics might still exist in statin users and nonusers, resulting in overestimating the benefits of statins on pneumonia hospitalization. For example, patients who adhered to statin therapy might have other related preventive behaviors (eg, exercising, avoiding tobacco and accessing medical help). Thus, rigorous and randomized controlled clinical trials are required to minimize the potential confounding bias and confirm the preventive effect of statin use on pneumonia hospitalization.

Our analyses showed some discrepancies about the recency of the benefits of statins compared with the previous studies. Nielsen demonstrated that statin use within 125 days before index pneumonia had a protective effect [10]. In another case-control study [11], Vinogradova demonstrated that the benefit was observed only in patients who used statin within 28 days before pneumonia. In the present study, we observed the protective effect on MI patients lasted for 90 days. The possible mechanisms underlying these discrepancies might be related to the differences of study designs, prescription rates of statin, commonly prescribed statins and patient characteristics between studies. Although discrepancies existed, the observations from our and previous research revealed that statin use might be associated with prevention of pneumonia in different patient groups.

Regarding prevention of adverse cardiovascular events, it remains controversial whether statin therapy before an MI event is beneficial. Some studies have reported that chronic statin pretreatment resulted in a small infarct area, more preserved ventricular function [7], and low hospital mortality [8] when patients developed acute MIs. However, Feurnau et al. recently showed that chronic statin pretreatment was not associated with minimal myocardial damage during MI events [20]. The differences of baseline risk profiles between patients exposed to statin pretreatment and those unexposed to pretreatment might have affected the effects of statin therapy [7, 8, 20]. Based on our results, statin use has superior benefits for patients unexposed to statin pretreatment than for all patients with MI. The major reason might be that MI patients exposed to statin pretreatment might have a significantly greater risk profile (e.g. hyperlimpidia) than patients unexposed to pretreatment [7, 8, 20]. Other potential reasons such as exact type, dose, duration and compliance of statin pretreatment might have also contributed to the heterogeneity [20]. These findings provide implications for further research on the pleiotropic effects of statins.

In our stratified analysis, statin users who were female or had fewer comorbidities had a significantly low risk of pneumonia. Our observations are similar to those of van de Garde, who reported that statin use was associated with a reduced risk of pneumonia in patients with diabetes who were female and had no previous pulmonary diseases [12]. Both findings suggested that the clinical presentation of pleiotropic effects of statins might be influenced by the individual’s risk profiles of patients. The specific conditions that might affect the effects of statins on the occurrence of pneumonia provide crucial information on how a randomized controlled trial should be designed to clarify the role of statins as an adjunctive treatment for the prevention of pneumonia in patients with MI.

In addition to time-dependent effect of statin use, our results showed a dose-dependent relationship of the benefits of statins that were similar to those reported by Wang et al., who demonstrated that statins prescribed at a medium or high DDD led to a 40–67 % reduction in the risk of COPD exacerbation, including concurrent pneumonia [21]. Additionally, two randomized controlled trials showed that statins exerted an anti-inflammatory effect in a dose-dependent manner [22, 23]. These findings raise a concern of optimal dosage of statins regarding their anti-inflammatory and pleiotropic effects. The exact mechanisms behind the effect of statin use on reducing the occurrence of pneumonia have not been completely elucidated. Statins have been shown to exert variable antimicrobial activity against various bacterial strains [24] and control the bacilli burden by enhancing host-induced autophagy and phagosomal maturation during pathogen invasion [25]. In addition, statins promote the clearance of microparticles from lung tissues to regional lymph nodes, attenuate recruitment and activation of alveolar macrophages, thereby reducing local proinflammatory cytokine production in the lungs [26]. The results from these aforementioned studies might provide possible mechanisms for the protective benefits of statin therapy. It still remains unclear whether different lipid-lowering abilities of statins can be correlated to their different anti-inflammatory abilities. In the present study, we observed that atorvastatin, fluvastatin and simvastatin had similar effects on reducing the risk of pneumonia among MI patients. Our findings were consistent with those of Vinogradova, who demonstrated that atorvastatin and simvastatin had similar associations with the risk of pneumonia in the general population [11]. These observations might provide implications for further clinical trials to compare the effects of different statins on the risk of pneumonia.

We observed that the percentage of any use of statins after MI in present study was only 60–70 %. Another study which was also conducted from an administrative database in the United States showed that 46.9 % of patients with high risk of cardiovascular disease were no longer taking a statin at a mean time of 3 months after drug initiation [27]. Adherence rates of statins in most observational studies have been low, with approximately 50 % at 6 months and 25 % at 1 year [28]. Even in clinical studies, adherence with statins is suboptimal, with 5-year discontinuation rate of 33 and 18 % in primary and secondary prevention trials, respectively [29, 30]. The most common reasons contributing to discontinuation and non-adherence are statin-related muscle side effects [31]. To increase the adherence rate and benefits of statins, especially in patients with MI and high risk of cardiovascular disease, statins should be prescribed after thoroughly evaluating the patient’s comorbidities, types and dosage of statins, and concomitant medications [31]. In this study, we considered the risk of COPD and found that COPD was associated with an increased risk of pneumonia requiring hospitalization (crude OR = 3.43, 95 % CI = 2.82–4.18, *P* < 0.001). The adherence rate of inhaled therapy in patients with COPD has been reported about 41.357 % and underuse of medications was common [32–34]. We did not analyze the adherence of medications for COPD because the numbers of MI patients with coexisting COPD were relatively small. Further clinical research is needed to investigate whether concomitant adherence to statins use and COPD treatment in MI patients with COPD could be an effective treatment in reducing the incidence of pneumonia requiring hospitalization.

### Limitations

Our findings are subject to certain limitations. First, the data on drug exposure were based on prescription claims, which might not reflect actual use. Second, the NHIRD does not have certain patient information, such as tobacco use and ambulatory status, which might contribute to the occurrence of pneumonia. Third, the NHIRD was derived from the administrative claims database that did not consider certain clinical information, such as the severity and etiology of pneumonia. Fourth, we did not further investigate the effects of specific statins. Fifth, we did not analyze the effect of concomitant use of statins and medications for coexisting pulmonary comorbidities. Finally, this study was conducted using a cohort of Taiwanese patients. The results might not be generalized to other populations. Future prospective, randomized studies on the effects of statins are warranted to confirm our findings.

## Conclusions

Statin use among patients with MI might be associated with a decreased risk of hospitalization for pneumonia in time- and dose-dependent manners, particularly for patients who were unexposed to statin pretreatment or had fewer comorbidities. Our results suggest that statin use might be used in an adjunctive therapy in preventing pneumonia hospitalization for patients with MI under thorough evaluation of individual comorbidities, previous statin use and optimal dosage.

## Additional files

Additional file 1: Table S1. Anatomical therapeutic chemical (ATC) classification system codes for drugs and defined daily dose (DDD) for statins. (DOC 32 kb) Additional file 2: Table S2. International classification of diseases, ninth revision, clinical modification (ICD-9-CM) codes. (DOC 31 kb) Additional file 3: Table S3. Title of data: Anatomical therapeutic chemical (ATC) classification system codes for drugs. (DOC 31 kb)

## Abbreviations

ACEI/ARBs: Angiotensin converting enzyme inhibitor and angiotensin receptor blockade
ATC: Anatomical therapeutic chemical
CHF: Congestive heart failure
CIs: Confidence intervals
CKD: Chronic kidney disease
CLD: Chronic liver disease
COPD: Chronic obstructive pulmonary disease
DDD: Defined daily dose
DDD_90_: Defined daily dose within a 90-day period
DM: Diabetes mellitus
HMG-CoA: 3-hydroxy-3-methylglutaryl-coenzyme A
HTN: Hypertension
ICD-9-CM: International Classification of Diseases, Ninth Revision, Clinical Modification
MI: Myocardial infarction
NHI: National Health Insurance
NHIA: National Health Insurance Administration
NHIRD: National Health Insurance Research Database
ORs: Odds ratios
PPIs: Proton pump inhibitors
