# Supplementary material for: Statin use associated with a reduced risk of pneumonia requiring hospitalization in patients with myocardial infarction: a nested case-control study
Source: BMC Cardiovasc Disord. 2016 Jan 28;16:24. doi: 10.1186/s12872-016-0202-x (PMC4730715; doi:10.1186/s12872-016-0202-x)
Supplement: Additional file 1: Table S1. — Anatomical therapeutic chemical (ATC) classification system codes for drugs and defined daily dose (DDD) for statins. (DOC 32 kb) [file 12872_2016_202_MOESM1_ESM.doc]

**Additional file 1: Table S1 Anatomical therapeutic chemical (ATC) classification system codes for drugs and defined daily dose (DDD) for statins.**

| **ATC code** | **Name** | **DDD** | **Unit** |
| --- | --- | --- | --- |
| C10AA01 | simvastatin | 30 | mg |
| C10AA02 | lovastatin | 45 | mg |
| C10AA03 | pravastatin | 30 | mg |
| C10AA04 | fluvastatin | 60 | mg |
| C10AA05 | atorvastatin | 20 | mg |
| C10AA06 | cerivastatin | 0.2 | mg |
| C10AA07 | rosuvastatin | 10 | mg |
| C10AA08 | pitavastatin | 2 | mg |
