# Supplementary material for: Statin use associated with a reduced risk of pneumonia requiring hospitalization in patients with myocardial infarction: a nested case-control study
Source: BMC Cardiovasc Disord. 2016 Jan 28;16:24. doi: 10.1186/s12872-016-0202-x (PMC4730715; doi:10.1186/s12872-016-0202-x)
Supplement: Additional file 2: Table S2. — International classification of diseases, ninth revision, clinical modification (ICD-9-CM) codes. (DOC 31 kb) [file 12872_2016_202_MOESM2_ESM.doc]

**Additional file 2: Table S2 International classification of diseases, ninth revision, clinical modification (ICD-9-CM) codes.**

| **Code** | **Description** |
| --- | --- |
| 410–414 | Ischemic heart disease |
| 410 | Myocardial infarction |
| 430–438 | Cerebrovascular disease |
| 250 | Diabetes |
| 401–405 | Hypertension |
| 272 | Dyslipidemia |
| 585 | Chronic renal disease |
| 428 | Heart failure |
| 491, 492, 494, 496 | Chronic obstructive pulmonary disease |
| 480–486 | Pneumonia |
| 493 | Asthma |
| 571 | Chronic liver disease |
| 332 | Parkinson disease |
| 290–294 | Dementia |
| 140–239 | Neoplasm |
