# Supplementary material for: Statin use associated with a reduced risk of pneumonia requiring hospitalization in patients with myocardial infarction: a nested case-control study
Source: BMC Cardiovasc Disord. 2016 Jan 28;16:24. doi: 10.1186/s12872-016-0202-x (PMC4730715; doi:10.1186/s12872-016-0202-x)
Supplement: Additional file 3: Table S3. — Title of data: Anatomical therapeutic chemical (ATC) classification system codes for drugs. (DOC 31 kb) [file 12872_2016_202_MOESM3_ESM.doc]

**Additional file 3: Table S3** **Anatomical therapeutic chemical (ATC) classification system codes for drugs.**

| **Category** | **ATC code** |
| --- | --- |
| ACEI/ARB | C09AA; C09BA; C09BB; C09CA; C09DA; C09DB; C09DX |
| Antiplatelet agent | B01AC |
| Nitrate | C01DA; C01DX |
| Statin | C10AA |
| PPI | A02BC |
| Systemic corticosteroid | H02A; H02B |
| Antineoplastic agents | L01A; L01B; L01C; L01D; L01X |
| Immunosuppresant | L04AA; L04AB; L04AC; L04AD; L04AX |
| Immunostimulant | L03AA; L03AB; L03AC; L03AX |
| Antiviral agents | J05 |

ACEI/ARB=angiotensin-converting enzyme inhibitor/angiotensin receptor blockade; PPI=proton pump inhibitor.
